# Retinal organoids: current status of development and new avenues for application in disease modeling, drug discovery and therapeutics

**DOI:** 10.1186/s40942-026-00846-x

**Published:** 2026-05-02

**Authors:** Renu Agarwal, Igor Iezhitsa, Jose R. Hombrebueno, Puneet Agarwal

**Affiliations:** 1 School of Medicine, International Medical University, Bukit Jalil, Kuala Lumpur, Malaysia; 2 Department of Inflammation and Ageing, School of Infection, Inflammation and Immunology, University of Birmingham, Birmingham, UK

**Keywords:** Retinal organoids, Pluripotent stem cells, Retinal diseases, Age-related macular degeneration (AMD), Retinitis pigmentosa (RP), Leber congenital amaurosis (LCA), Drug discovery, 3-D cell culture, Gene therapy, Personalized medicine

## Abstract

Visual impairment affects over 2.2 billion people worldwide and the major causes include age-related macular degeneration (AMD), glaucoma, and diabetic retinopathy. For research in these areas, although animal models offer a more physiologically complex system than in vitro approaches, their use raises ethical considerations, and species-specific differences such as variations in protein sequences and signaling pathways. This can limit the direct translatability of the outcomes. Traditional 2-D cell cultures, in contrast, lack the multicellular organization and dynamic microenvironment necessary to replicate human retinal complexity. Retinal organoids (ROs), three-dimensional tissue constructs derived from pluripotent stem cells, have emerged as a promising model due to their human origin and complex cellular interactions that cannot be achieved in conventional 2-D/3-D co-culture models. In this review, we provide a brief overview of the evolution from 2-D to 3-D retinal models, highlight the structural and functional features of ROs including the presence of layered retinal architecture, photoreceptor outer segment formation, and light-responsive electrophysiological activity and summarize their applications in disease modeling, drug discovery, and gene and cell therapy. ROs represent a significant advancement over traditional models by enabling the recapitulation of human-specific retinal development, facilitating the study of patient-derived disease phenotypes, and providing a platform for personalized therapeutic screening. Their development has deepened understanding of pathological mechanisms in conditions such as retinitis pigmentosa and AMD, while enabling preclinical testing of targeted interventions like CRISPR-based gene editing and photoreceptor cell replacement. Nonetheless, challenges remain in fully replicating retinal vascularization, long-term functional maturation, and synaptic connectivity, underscoring the need for continued refinement and integration with complementary model systems.

## Highlights

Retinal organoids (ROs), have emerged as a critical platform for advancing our understanding of retinal diseases and drug discovery, with the potential to overcome key limitations of traditional 2-D cell cultures and animal models.

ROs offer increased structural and cellular complexity compared to traditional 2-D cultures, providing a more physiologically relevant system to study human retinal biology under physiological and pathological conditions.

Since ROs can be derived from patient-specific pluripotent stem cells, they hold promise for enabling the development of personalized therapeutic strategies and enhance our understanding of gene and cell therapies.

Despite promise, they still face important limitations, including incomplete neuronal connectivity, inconsistent cell composition, long maturation times, and reduced long-term viability.

## Introduction

According to the World Health Organization, approximately 2.2 billion people have a near or distance vision impairment, globally, and the annual global cost of productivity is estimated to be US$ 411 billion [1, 2]. Besides refractive errors and cataract, leading causes of visual impairment include age-related macular degeneration (AMD), glaucoma, diabetic retinopathy and inherited photoreceptor degenerations [1]. These ocular diseases are driven by complex and heterogeneous pathogenic mechanisms, which contribute to the limited efficacy of current therapies and present significant challenges for the development of new therapies. Traditionally, preclinical research has largely utilized 2-D cell culture systems and animal models, both with inherent limitations. 2-D cell culture systems lack the complex in vivo physiological environment consisting of diverse cell types, tissue architecture and intercellular signaling dynamics, both mechanical and biochemical. Among various animal models, rodents have particularly been popular for investigational purposes. Laboratory mice have a short lifespan but recapitulate disease features that take years to progress in humans. They are cost-effective and genetic studies in rodents have proved particularly useful for exploring disease-causing variants and pathological mechanisms due to similarity in the genetic background of humans and rodents. However, animal models pose other key limitations [3] such as no identifiable mouse homologues for about 1% of human genes and possibility of different copy numbers for the remaining 99% of homologous protein coding genes [4, 5]. Failure to accurately reproduce features of human disease in rodents also emerges from anatomical differences. For example, rodents do not have macula or fovea, which is the affected region in several retinal diseases in humans. Consequently, significant differences in drug efficacy and toxicity outcomes may arise in animal studies due to inadequate disease modeling.

An ideal approach to modeling human retinal disease involves incorporating all major retinal cell populations into a 3-D tissue construct that closely mimics the vivo microenvironment. New technical advances have hugely contributed to developing tissue constructs with advanced biomimicry that can be subjected to experimental evaluations with greater translational value [6–8]. Retinal organoids (ROs) have emerged as powerful tools with unparalleled potential for modelling human retinal cytoarchitecture and function, ex-vivo. Therefore, they provide a promising platform with advanced solutions to current deficiencies in research related to retinal diseases and therapeutics.

In this review, we explore the literature published over the past decade to understand the application of ROs for drug discovery. Firstly, we present an overview of the evolution of ROs from 2-D cultures to 3-D tissue constructs. Then we examine the structural and functional attributes that confer ROs their utility in specific investigative contexts. Finally, we highlight the current applications of ROs in disease modeling, drug discovery, gene therapy, and cell therapy, and briefly address their limitations and advances in next-generation ROs. The literature search was done using PubMed search engine using key words such as retinal organoids, pluripotent stem cells, retinal diseases, gene therapy, transplant, drug screening, photoreceptors, retinal ganglion cells (RGCs), retinal pigment epithelium (RPE) in various combinations. Papers published during 1997 till 2025 are included in this review.

## Development of retinal organoids from 2-D to 3-D: a brief overview

Organoids are in vitro 3-D representations of specific organs and are derived from pluripotent stem cells (PSC). Currently, ROs are most commonly derived from embryonic stem cells (ESC), induced PSCs (iPSC), retinal progenitor cells (RPC), and other types of stem cells from humans and rodents [9–11]. Among all, iPSCs are one of the most reliable and widely used for deriving ROs. For example, a recent report by Capowski et al. showed the successful development of ROs from 16 different human PSC (hPSC) [10].

**Fig. 1:**
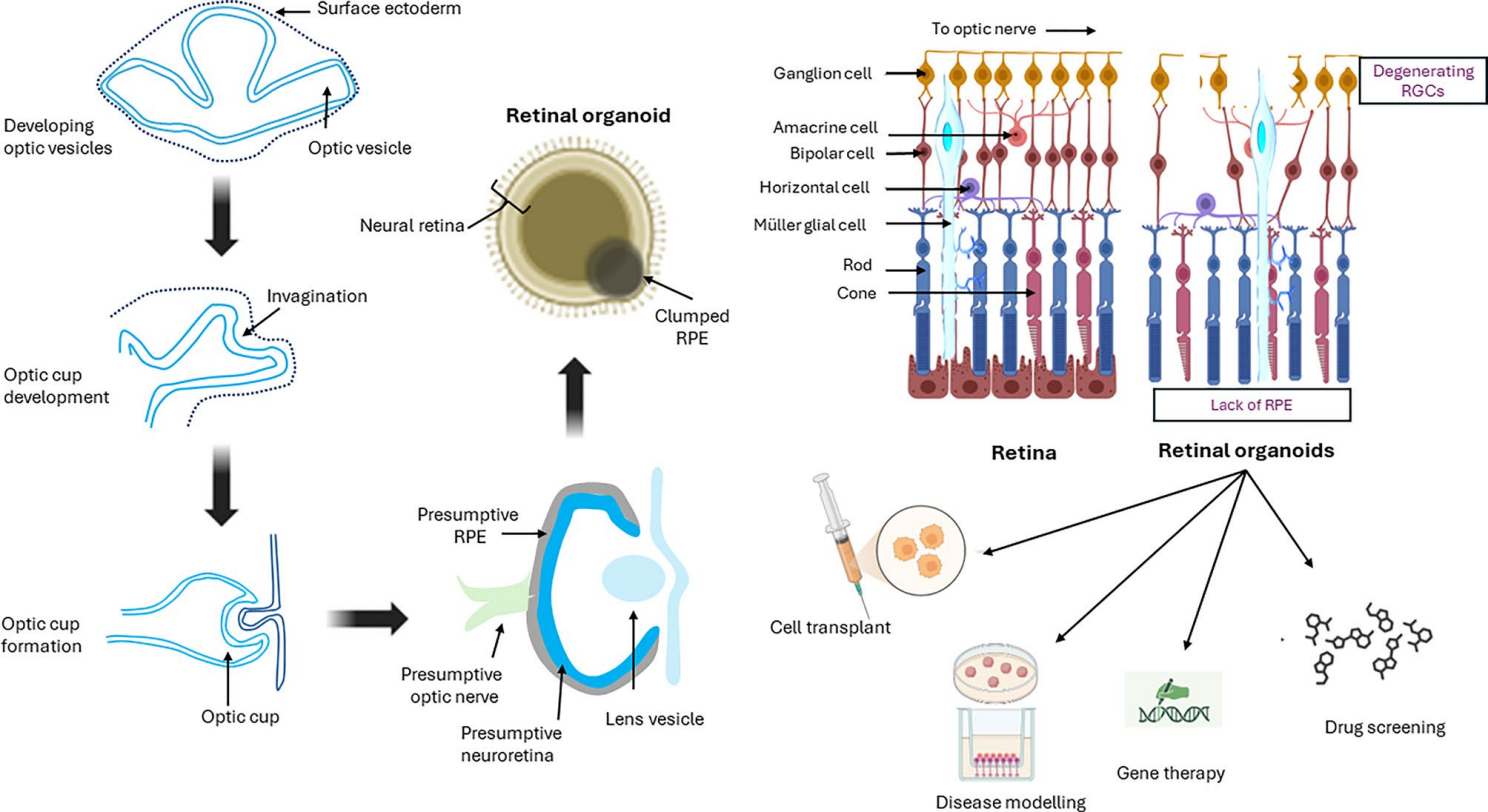
Diagrammatic representation of the development of retinal organoids from optic vesicles. Figure also depicts the histomorphological differences between retina and retinal organoids and potential uses of retinal organoids. RPE: retinal pigment epithelium; RGCs: retinal ganglion cells. The figure has been prepared with the help of biorender.com

In the fetal eye, the retina develops from neuroectoderm. During embryonic development, the forebrain divides into the telencephalon and diencephalon. A pair of optic vesicles develops from the ectoderm in the eye field regions of the diencephalon. Subsequently, the distal part of optic vesicles invaginates to form double-layered optic cups. The invagination occurs in an apically convex manner that is reflective of an intrinsic biomechanical remodeling capacity of developing tissue and mimics in vivo embryonic retinogenesis [12, 13]. The outer layer differentiates into RPE and the inner wall generates neuroretina [14]. Importantly, ROs derived from PSC recapitulate the key stages of fetal retinal development, which includes sequential emergence of major retinal cell types, spatial organization into layered neuroepithelium, and photoreceptor maturation (Fig. 1).

Initial studies applying principles of classical developmental biology laid the foundation for the development of the 3-D ROs widely used today. Observations that forebrain development could be induced in mice by inhibiting Wnt and BMP signaling, and in *Xenopus* embryos by injecting insulin-like growth factor 1 (IGF1), guided the development of 2-D ROs. Accordingly, 2-D differentiation of PSCs into retinal lineages was achieved by incorporating a Wnt inhibitor (Dickkopf1; DKK1), a Bone Morphogenic Protein (BMP) inhibitor (Noggin), and IGF1 supplementation [15–18]. While these early 2-D models successfully generated rhodopsin+ photoreceptors from mouse ESCs (mESCs), the laminar retinal organization characteristic of in vivo retina could not be generated.

Further development of ROs to desirable structures is shaped by significant discoveries in stem cell biology and developmental neuroscience. Meyer et al. first demonstrated that hiPSCs could differentiate into primitive anterior neuroepithelium, which in turn gave rise to either RPCs or early forebrain cells [19]. Around the same time, studies using hESCs revealed the emergence of two distinct cell populations after 20–25 days of culture: vesicular structures expressing RPE-specific transcription factors and non-vesicular aggregates expressing early forebrain markers [20]. Eiraku et al. successfully generated a 3-D optic cup and stratified neuroepithelium from mPSCs, mimicking the key morphological events of in vivo embryonic retinogenesis [12]. In their protocol, embryoid bodies (EBs) derived from mPSCs were cultured under low-growth factor conditions with Matrigel used as an extracellular matrix (ECM) supplement. Matrigel, rich in laminin, collagen, and epidermal growth factor (EGF), supported the self-organization of optic vesicle structures, leading to spontaneous differentiation into RPCs and further maturation into RPE and neuroretina. The resulting 3-D lamellar structures contained all six major neuronal cell types of the retina, along with Müller glial cells.

Building on these findings, Reichman et al. later developed a more efficient protocol that bypassed the EB stage, enabling faster generation of RPE and neural retina from hiPSCs within two weeks [11]. Subsequent studies further demonstrated that hiPSCs can undergo spatiotemporal differentiation that closely recapitulates each stage of in vivo retinal development, ultimately producing 3-D retinal cups composed of all major retinal cell types arranged in correct laminar organization [21]. Importantly, the commitment of pluripotent stem cells toward either the neuroretinal or RPE lineage was found to be governed by the co-expression and spatial localization of two key markers at the optic vesicle stage: visual system homeobox 2 (VSX2), specific for neuroretina, and microphthalmia-associated transcription factor (MITF), specific for RPE. At the optic cup stage, VSX2 and MITF localize to the presumptive inner neuroretina and outer RPE, respectively, reflecting the early lineage segregation observed during normal retinal development [22, 23].

Currently, the protocols used to generate ROs from PSCs follow a common core developmental framework across studies [9, 10, 12, 21, 24–27]. Most protocols begin with well-characterized hESC or hiPSCs maintained under serum-free, feeder-independent conditions, typically using defined media such as mTeSR1 or Essential 8 on ECM substrates including Matrigel or vitronectin to ensure homogeneous pluripotent cultures. Early differentiation into EBs is initiated by transferring dissociated cells or small cell aggregates into low-adhesion 3-D culture systems most commonly in U- or V-bottom multi-well plates. EBs are then exposed to neural induction conditions, usually consisting of DMEM/F12 supplemented with N2 and/or B27, with or without vitamin A (retinol). While retinol is usually avoided in the very early stages to prevent premature differentiation, it is critical in the late stages for photoreceptor maturation. Suppression of non-neural signaling pathways, particularly BMP and TGF-β signaling, is widely used to guide differentiation toward anterior neuroectodermal fate. While initial BMP inhibition is common, some protocols actually use a BMP4 pulse later (around day 6) to enhance the neural retina differentiation. During this early patterning phase, key transcription factors including PAX6, RAX, LHX2, OTX2, and VSX2 are expressed indicating acquisition of eye-field identity. Continued 3-D growth leads to spontaneous evagination of neuroepithelial domains to form optic vesicles and then invagination to generate optic cup-like structures. These structures give rise to distinct neural retina and RPE domains, typically within the first month of differentiation. To stabilize epithelial polarity and support optic vesicle morphogenesis, some protocols incorporate transient attachment of aggregates to ECM substrates, whereas others rely on free-floating self-organization. Optic vesicles or optic cup-like structures are often manually isolated and cultured to develop ROs which are then maintained in retinal differentiation media. Long-term maturation of ROs with appearance of postmitotic retinal cells and laminar organization proceeds over weeks to months. Photoreceptor differentiation and outer segment maturation are commonly promoted through temporally controlled addition of factors such as retinoic acid, thyroid hormone, taurine and modulation of Notch signaling.

To achieve desirable experimental conditions including accelerated maturation, improved reproducibility, incorporation of non-neuronal cell types, and modeling of retinal disease states several variations have been introduced to the core framework. These modifications primarily aim to optimize metabolic support, oxygen availability, and dynamic culture conditions to enhance tissue viability and functional maturation (Table 1). One of the approaches was to culture hiPSC on Matrigel-coated culture dishes in a chemically defined neural differentiation medium. It led to development of aggregates expressing neuroepithelial markers, PAX6 and SOX1, by day 8. Subsequently, by day 12, there was gradual loss of pluripotency, acquisition of neural fate and the progressive differentiation into retinal progenitors in the eye field like domains mimicking in vivo embryonic development. Next, the optic vesicles developed with retinal progenitors eventually forming the neuroretina and RPE. Hence, in this study, 3-D structures not only possessed spatial and temporal features similar to the in vivo human retinal development, they also showed presence of all retinal cell types including RGCs, amacrine, bipolar, horizontal, rods, three types of cones and Müller glial cells in properly arranged layers. However, compared to photoreceptors, the RGCs were progressively lost during long-term culture [21]. In another method, clumps of matrigel adherent human ESC (hESC) were cultured to obtain single lumen epithelialized cysts, which developed patterned retinal monolayers with tight junctions. Subsequent floating culture led to self-development of ROs consisting of neuroretina, ciliary margin, and RPE [29]. Using an alternative approach with a modified xeno-free/feeder-free culture media, hiPSC were shown to undergo spontaneous differentiation, neural induction, neuroepithelial formation and development of 3-D ROs after growing to confluence without EB formation [33, 35]. To efficiently generate cones suitable for isolation and transplantation, hPSCs were cultured in fibroblast growth factor (FGF)-free medium for two days, followed by neural induction. From weeks 4 to 7, isolated neuroretinal vesicles were maintained in retinal differentiation medium, with fetal bovine serum, taurine, and GlutaMAX added at week 6, and retinoic acid introduced at week 10. For long-term culture, retinoic acid levels were reduced and N2 supplement was added. By weeks 3–4, lightly pigmented RPE clusters appeared, followed by optic vesicle-like structures containing neuroretinal vesicles with retinal neuroepithelium. Remarkably, all neuroretinal vesicles expressed photoreceptor markers, mirroring fetal photoreceptor development. For instance, an ONL-like layer formed at week 15, similar to its emergence at week 14 in fetal retina. When transplanted into Aipl1−/− mice, these cells formed synapse-like structures with host interneurons and expressed photopigments [32]. Similarly Kim et al. used an improved ECM–induced retinal differentiation system to generate cone-rich ROs from hESCs [44]. The bulk RNA‑seq and 8‑month single-cell RNA‑seq, demonstrated that in vitro development mirrored in vivo retinogenesis, confirming the presence of distinct cone and rod clusters. Importantly, organoid‑derived cones exhibited electrophysiological function and possessed transcriptomes closely matching those from the human macula [44]. Gao et al. used patient-iPSC-to derive ROs harboring a PDE6B mutation to model late-onset retinitis pigmentosa (RP) [62]. A remarkably distinct gene expression profile was observed in the patient ROs at differentiation day 230. The protocol included gradual and timed supplementation with hBMP4 on 6th, 9th and 12th day of culture, non-adherent culture at day 18 followed by long-term culture with neural retina medium refreshed with retinoic acid and other supplements, every 5 days, without physical isolation of optic vesicles. This approach minimized manual handling and allowed for generation of ROs with enhanced throughput and consistency [62]. Regent et al. described a protocol in which traditional all‑trans retinoic acid was replaced with 9‑cis retinal [47]. This method significantly accelerated photoreceptor development, improved outer segment morphogenesis with longer photoreceptor outer segments and gave higher RO yield [47]. Li et al. used a whole culture scraping method which involved scraping off the entire adherent stem cell monolayer rather than manually dissecting optic vesicles followed by transition into 3-D suspension [49]. The method simplified the workflow and was reported to increase RO yield by up to 5‑fold [49].

**Table 1:** A summary of protocol modifications used over last decade to obtain retinal organoids and the benefits and limitations of these modifications

Reference	Type of cells used	Key protocol modification	Benefits of modified protocol	Limitations of protocol
Chen et al. [28]	mESCs and mPSCs	Hypoxic conditions (low O₂) were applied during initial culture (day 0-day10) to mimic in vivo low-oxygen environment.	Higher efficiency in generating optic vesicles (OV) and optic cups (OC) compared to normoxic conditions; relatively larger OV and OC; stratified neural retina (NR) with all major cell types including photoreceptors with ciliary growth and synaptogenesis by day 35.	Inherent variability in response to hypoxia across cell lines leading to variability in the yield and quality of ROs across different batches and cell lines; protocol is specific to mouse stem cells.
Lowe et al. [29]	hESCs and hiPSC	Addition of blebbistatin and Y27632 to the culture medium 1 hour before and during dispase treatment, and during culture. Instead of manual dissection or mechanical scraping dispase was used to detach adherent culture during days 12–17 and clumps were cultured in a floating system to generate epithelialized cysts.	Larger yield of OC for culture with minimal manual manipulation; More efficient epithelialization compared to solidified layers.	Potential variability in detachment efficiency, RO structure and functions require further validation to ensure reproducibility and consistency.
Völkner et al. [30]	mESC and hESC	Dissection based on classic OV evagination was replaced by mechanically trimming aggregates into retinal domains.	Increased efficiency in terms of the yield of stratified ROs per differentiation; improved reproducibility and temporal control of retinogenesis.	Labor-intensive and operator-dependent; batch-to-batch variability, morphology may differ slightly from OV evagination.
DiStefano et al. [31]	mESC and miPSC	Use of a rotating-wall vessel (bioreactor) to culture dissected NR from ROs rather than static suspension to improve mass transfer of oxygen/nutrients and promote biophysical stimulation.	Enhanced growth and differentiation kinetics, improved lamination and neuronal differentiation across cell lines compared to static culture.	Bioreactor setup cost, complexity and sensitivity to shear stress; heterogenous nutrient distribution especially to inner layers; risk of potential oxidative stress; limited functional maturation beyond early postnatal stages.
Gonzalez-Cordero et al. [32]	hPSCs	A combination of 2-D/3-D differentiation protocol. OV-like structures containing future NR vesicles appear in 3–4 weeks of differentiation. NR vesicles were manually dissected along with small amounts of retinal pigment epithelium (RPE) and subjected to suspension culture in media containing fetal bovine serum, taurine, and retinoic acid.	Sufficiently high number of photoreceptors, especially cones with transplantation potential; maturation of later stages of photoreceptors is promoted; cone and rod differentiation closely resembles human retinal development.	Lack of close opposition to RPE and tight stacking of outer segment membrane discs; presence of synaptic connections and functional activity of photoreceptors remains uncertain.
Reichman et al. [33]	hiPSCs	Use of a xeno-free/feeder free culture media. iPSCs were adapted to feeder free conditions and cultured in dishes coated with truncated recombinant human vitronectin (rhVTN-N) in Essential 8 medium.	Method bypasses embryoid body (EB) formation; transplantation-compatible CD73+ photoreceptor precursors could be generated in less than 100 days; mature cones and rods with specific ultrastructure could be maintained until 280 days; both the dissociated retinal cells and ROs could be cryopreserved with retention of phenotypic features and CD73+ photoreceptor precursors.	Potential variability across cell lines; efficiency may be affected due to lack of animal derived growth factors; protocol may not be easily scalable for large-scale production; long-term viability and functionality of stored ROs needs validation.
Wahlin et al. [34]	hPSCs	Protocol includes manual picking of neuroepithelial clusters from day 28–35; retinal differentiation medium (RDM) enriched with fetal bovine serum, taurine, and retinoic acid used for suspension culture after day 30 followed by long-term culture up to 200 days.	Development of photoreceptors with outer segment-like and calyceal processes like structures; protocol validated for16 different hPSCs.	Labor-intensive due to reliance on manual picking; incomplete lamination; misplaced photoreceptors likely in the inner layers; lack of functional RGCs.
Eldred et al. [35]	hPSC	Triiodothyronine (T3) was added starting at the time corresponding to cone differentiation (day 50 onwards).	Protocol mimics thyroid hormone signaling dynamics during in vivo retinal development; presence of physiologically relevant cone subtypes ratios.	Strict control of dose and timing for T3 treatment is required; extended time required for culture; in vivo environment is only partially recapitulated as systemic factors other than T3 are not present; limited functional validation; potential variability across cell lines.
Fligor et al. [36]	hPSCs	Cell aggregates were plated onto coverslips coated with poly-ornithine and substrates such as laminin; addition of growth factors such as BMP2, BMP13, NT4/5, GDF8, BDNF, or netrin-1 to laminin-coated coverslips in RDM.	ROs have a defined presumptive RGC layer within 70 days of differentiation; the developmental timing and organization of the RGCs is similar to native retina making it more physiologically relevant; RGC neurite outgrowth is significantly enhanced by netrin-1.	Extended culture duration, complexity of protocol; batch-to-batch variability; RGCs may lack complete functional synaptic connections.
Hallam et al. [37]	hiPSCs	The protocol employs culture under hypoxic conditions (5% O2) during first 7 days of early differentiation after which, normoxia (20% O2) is restored for long-term maturation.	Protocol mimics the physiological hypoxic environment of the early embryo; hypoxia enlarges the pool of retinal progenitor cell available for differentiation; significant enrichment with photoreceptors; useful for modeling conditions such as retinopathy of prematurity.	Requirement for specialized chambers for hypoxia exposure; tight control of hypoxia duration to protect against inner-core necrosis; variations in hypoxic response across cell lines.
Li et al. [38]	hiPSCs	hiPSC were derived from urine; retinoic acid was not added to the culture medium throughout the differentiation	Non-invasive source of PSCs; development of mature rods and cones; reproducibility across multiple urine derived cell lines; culture duration could be extended beyond 120 days.	Extended differentiation and maturation timeline; variable differentiation efficiency and yield; functional validation will be required; differentiation may be affected by donor-specific epigenetic memory or genomic instability.
Achberger et al. [39]	hiPSCs	Protocol uses standard methods to generate ROs and RPE cells separately; ROs and RPE are then integrated on day 40 onwards into a polydimethylsiloxane (PDMS) membrane containing microfluidic chip; to mimic blood flow, media is continuously perfused through the chip’s “vascular channel”.	Physical interaction between RPE and photoreceptors is enabled; method allows controlled perfusion and nutrient supply.	Cost and complexity associated with use of specialized microfluidic equipment; compared to standard well-plate culture method gives a low throughput.
Brooks et al. [40]	hiPSC	Addition of docosahexaenoic acid (DHA) in cultures from day 10–32; Addition of fibroblast growth factor-1 (FGF-1) from day 26–32.	DHA-treated ROs had a 30% increase in rhodopsin expression and enhanced ciliogenesis on day 32; more developed photoreceptor inner segments with mature mitochondria. FGF1-treated ROs showed significantly greater expression of cone-related genes, longer cilia and outer segment spirals at day 32.	Lack of complete photoreceptor outer segments and synapses; lack of functional maturation; short term viability as ROs degenerate after day 32.
Capowski et al. [10]	hPSC	The differentiation was done on an adherent monolayer. Specific vesicular neuroepithelial structures were identified and manually dissected to create free-floating 3-D organoids. All-trans retinoic acid was added to RDM to enhance photoreceptor outer segment formation.	High reproducibility across ROs developed from 16 cell lines; The protocol reduced contamination with the forebrain or non-neural tissue.	Labor intensive; variable efficiency in terms of number of ROs produced per starting well; long maturation time; less suitable for high-throughput.
Chichagova et al. [41]	hPSC	Protocol uses single-cell dissociation and seeding on 96-well Lipidure-coated plates with air-drying for 24 hours before use compared to standard protocols that use low attachment plates or extracellular matrix (ECM)/Matrigel embedding; IGF-1 is added to medium from differentiation day 3 onwards; FBS, T3 and taurine added from day 18 onwards; culture maintained for >22 weeks.	Stage-specific use of media to guide differentiation and maturation; large-scale RO production is likely as cells differentiate in 96-well plates; stratified neuroepithelium after 22 weeks with photoreceptors, bipolar, horizontal, amacrine, Muller, and retinal ganglion cells (RGCs).	Variability in size and composition; complexity of various differentiation protocols; long-term culture requires validation.
Dorgau et al. [42]	hPSC	Peptides from decellularised ECM from NR and RPE were added to culture media after day 18 of differentiation.	More natural development and enhanced maturation; enhanced expression of synaptic markers indicating improved synaptogenesis; improved functionality indicated by light responsiveness.	Potential variability due to limited information on the peptide composition; batch-to-batch consistency and reproducibility cannot be ensured; potential immunogenic risks.
Kaya et al. [43]	hESCs and hiPSC	Addition of 9-cis-retonoic acid to photoreceptor induction medium from days 63 – 92 at a concentration of 1 µM followed by 0.5 µM.	Rod photoreceptor differentiation accelerated with increased rhodopsin expression and mitochondrial maturity was evident by day 120.	Limited photoreceptor outer segment formation and synaptic connectivity; instability due to light sensitivity of 9-cis retinoic acid; further need for functional validation.
Kim et al. [44]	hESC and hiPSCs	Cell aggregates were cultured in neural induction medium (NIM) to drive early EB differentiation toward a retinal progenitor identity. OVs and adjacent RPE were manually picked for further culture; RDM used after day 90 was devoid of retinoic acid hence driving cone differentiation.	Production of cone-rich ROs that resemble the human macula/fovea based on cone: rod ratio and molecular expressions. Lack of retinoic acid seems to provide a permissive condition for cone differentiation.	The cell composition in ROs after long-term cultures differs from the macula; photoreceptor outer segments did not reach maturity; though these ROs resemble macula, they could not be considered equivalent.
Kuwahara et al. [45]	hESCs and hiPSC	Preconditioning of hPSC colonies with SB431542, LDN193189 and/or smoothened agonist (SAG) for 18–30 h prior to differentiation. BMP4 was added on day 3 of SFEBq culture. Thereafter, BMP4 concentration was reduced to half.	Reduced intra- and inter-batch variability in RO size and morphology; preconditioning stimuli affect the NR morphology and control the proportions of NR and RPE in ROs; LDN-preconditioned hiPSCs tend to differentiate into NR dominant aggregates; SB-preconditioned ROs have NR-RPE conjugated aggregates; greater reproducibility; potential for genetic modification studies.	Potential variability in NR and RPE patterning; unclear molecular mechanisms; functional integration post-transplant remains challenging.
Cowan et al. [26]	hiPSCs	The protocol named AMASS (Automated Maintenance of Aggregates in Suspension Systems) utilized an automated, large-scale suspension culture in early phase; manual picking was replaced with high-density suspension in which neuroepithelium self-organizes into OV; culture period was extended to >250 days to allow adult-like molecular states.	ROs had three nuclear and two synaptic layers; a high degree of transcriptomic similarity of ROs with newborn human retina; reproducibility; temporal accuracy; high scalability.	Long-term maintenance and associated high cost.
Pan et al. [46]	hESC	Use of COCO, a multifunctional antagonist of the Wnt, BMP, and TGF-β pathways up to day 30.	Increased photoreceptor differentiation efficiency in the early stage. The enhanced photoreceptor precursor fate towards cones and decreased towards rods over a longer time-frame.	Decline in the number of rods and reduced RO survival; careful timing of COCO addition is required to avoid RO damage; possible reduction in photoreceptor maturity and functionality over long-term.
Regent et al. [47]	hESCs and hiPSC	A scraper was used to remove clumps of adherent cells on day 20–30. Clumps were maintained in free floating culture in RDM consisting of insulin-like growth factor (IGF) 1. From day 63 onward, 9-cis retinal, was added and its concentration was reduced to half from day 91 onwards.	A 5-fold greater yield of ROs post-scraping compared to traditional method across 6 cell lines; simpler method requiring less technical expertise and less labor intensive; less subjectivity compared to traditional method that requires selection of OV domains; more number of ROs consist of RPE domains; potential for large scale production.	Potential variations in size and maturity depending on the size of the scrapped clumps; lack of clear outer segment structures; may not fully recapitulate the functional maturity.
Zerti et al. [48]	hESC	Stage-specific addition of retinoic acid, 9-cis-retinal, 11-cis-retinal, triiodothyronine (T3), levodopa (l-DOPA), and γ-secretase inhibitor ((2S)-N-[(3,5-Difluorophenyl)acetyl]-l-alanyl-2-phenyl]glycine1,1-dimethylethyl ester2L [DAPT]) to direct the generation of the type of photoreceptors in ROs.	During differentiation, addition of retinoic acid with T3 from day 90 to 120 enhanced rod and S-cone generation; addition of DAPT (days 28–42) with retinoic acid (days 30–120), enhanced L/M-cones generation at the expense of rods; l-DOPA with retinoic acid (days 90–120) enhanced S-cones generation at the expense of rod photoreceptors.	Cell line dependent variabilities; impact on other retinal cell types, long-term stability and functional maturity remains unclear.
Li et al. [49]	hESC	SEAM (Self-formed Ectodermal Autonomous Multi-zone) culture system that omitted Wnt, BMP, or Nodal inhibitors was used; free floating 3-D aggregates were cultured under low-adhesion conditions to facilitate apicobasal self-organization and OC evagination; no exogenous activin, and retinoic acid was added at later stages.	Exogenous factors are not required, reducing variability; retinal differentiation resembles early and late differentiation in vivo; method also generates ectodermal derivatives (lens, cornea, RPE) enabling studies on tissue interactions; development of mature photoreceptors with rods distributed proximally and cones distally; potential scalability for production.	Requires precise control of initial seeding densities and sphere size; labor intensive; chances of batch-to-batch heterogeneity in morphology and cell-type ratios; microglial presence is not prominent; validation is required for other cell types and modeling of retinal diseases.
Wagstaf et al. [50]	hESCs	Suspension of cell clumps maintained in undiluted Matrigel followed by plating drops totally encasing the cell clumps in a matrigel environment; retinoid acid added from day 7–120 to RDM; on day 84 retinoid acid concentration was halved and T3 was added.	Use of Matrigel provided a 1.5-fold higher IGF-1 concentration at early developmental stage compared to conventional methods leading to a high yield of RGCs within 4 weeks without adding any external factors.	Long-term viability and longevity remains uncertain; stratification remains immature; development of other retinal cell types needs validation.
Chew et al. [51]	hESCs and hiPSC	Inhibition of Notch signaling pathway at different developmental time-points using a small molecule (PF-03084014) for 3 days starting at days 45, 60, 90 and 120	Early loss of progenitor properties; increased appearance of immature cones following days 45 and 60 treatment; increased appearance of immature rods following day 90 treatment; pool of immature photoreceptors may achieve better integration post-transplantation, compared to mature photoreceptors.	Lack of maturation, lamination and organization of the photoreceptors; lack of other native retina cell types.
Sanjurjo-Soriano et al. [52]	hiPSC	Modified culture medium supplementation with addition of taurine on day 42, retinoic acid along with B27 without Vit A on day 65, N2 supplementation on day 85 onwards, removal of retinoic acid on day 120.	Though the initial differentiation of photoreceptor is delayed, more structured photoreceptors appear at maturity; stratification and overall structure of mature ROs significantly improved; observed effects were primarily attributed to retinoic acid.	Delayed early differentiation; suppression of cone differentiation, requirement for multiple supplements with complex timing; labor intensive; limitation for foveal/macular disease modeling.
West et al. [53]	mESC and hPSC	Supplementation with antioxidants and DHA and other essential lipids starting on day 70 that corresponded to mid-late differentiation stage till later stages of RO maturation (day 120 and onwards) to promote photoreceptor maturation.	Significantly improved length, morphology and ultrastructure of photoreceptor outer segments; improved photoreceptor survival and light sensitivity; simple protocol.	Timing and concentration of multiple supplements needs optimization; cell-cell interactions may remain limited; functionality remains suboptimal; stability following transplantation remains uncertain.
Usui-Ouchi et al. [54]	hiPSCs	Macrophage precursor cells (MPCs) emerged in 4 weeks following EB culture in media containing interleukin-3 and macrophage colony-stimulating factor (M-CSF). For producing ROs separately, EBs were fragmented and scrapped on day 28 and subjected to suspension culture. At week 8, ROs were transferred to a bioreactor in RDM and co-cultured with MPC with gradual shift from 3:1 to 1:3 ratio of MPC media: RO media containing M-CSF. M-CSF was discontinued from 4 to 7 days of co-culture.	Controlled integration of microglia with ROs to model microglia/retina interactions; improved lamination and maturation.	Complex protocol with precise timing and media compositions; long overall timeline; need for specialized equipment (bioreactor); batch-to-batch variability.
Matsushita et al. [55]	hESC and hiPSC	For initial NR induction, dual SMAD inhibition (Noggin and SB431542) and BMP4 treatment was used as a SEAM method; for retinal differentiation SAG, activin A, and retinoic acid were used concurrently from day 10–40 and switching to SAG treatment alone for further stratification and maturation.	Efficient early neural induction and accelerated development of mature ROs within 90 days compared to 120 days with standard protocols; improved stratified and mature photoreceptor layer; prominent presence of other retinal cell types.	Method used specific cell lines developed in Kyoto University and those established by RIKEN, hence limited validation across cell lines; loss of RGCs by day 40; pharmacological complexity may lead to variations; further structural, molecular and functional characterization would be needed.
Harkin et al. [56]	hESCs and hiPSCs	Modification applied at early stage through dissociation of colonies into single-cell suspensions and generate aggregates of consistent size and shape. Cells were then seeded at defined cell numbers followed by forced reaggregation by centrifugation.	Nearly universal generation of ROs with high purity and consistency across cell lines; considerably reduced variability and enhanced reproducibility; expedited differentiation of cell types compared to traditional protocols.	Method requires precise cell counting and reaggregation control, which could limit flexibility and throughput; differentiation efficiency may vary depending on the quality of reagents, particularly recombinant BMP4.
Kawai et al. [57]	hPSC	Addition of hyaluronan (HA) treatment at 14 weeks of differentiation and continued till week 26. ROs with minimal RPE were selected to reduce variability caused by RPE-secreted HA. For other photoreceptor studies HA was added from 28 to 32 or 32–34 weeks.	ROs were smaller but more consistent in size; accelerated differentiation of retinal progenitor cells into photoreceptors; increased expression of photoreceptor genes and preserved retinal lamination; reduced brush border layer length; enhanced photoreceptor maturation.	Photoreceptors show structural differences after HA treatment; development of other retinal cell types and synaptic connections and effects of long-term culture remain unclear.
Xu et al. [58]	hESCs	Microglial differentiation promoted by culturing EBs on low adhesion plates; microglia harvested from EBs on day 56 and co-cultured with separately grown ROs on day 12 of their differentiation for 50 days.	Closer to physiological environment of cell interactions allowing studies related to cellular interactions and screening of drugs with mechanisms relevant to these cell interactions.	Complex experimental design; prolonged differentiation periods; meticulous time planning for separate and then co-culture of microglia and ROs; effective microglial cryopreservation is challenging; variable reproducibility.
Chen et al. [59]	iPSC	A separate differentiation track to generate vascular organoids (VOs); mature ROs are physically fused with VOs using a microwell system to produce vascularized ROs (vROs); fusion performed in shallow PDMS microwells, instead of deep 96-wells for precise microscopic alignment and more reproducible fusion; VEGF-supplemented retinal maturation medium used for culture after fusion.	Development of vascular structures inside ROs after 30–120 days of co-culture; microglia from VOs integrate into vROs enabling an inflammatory responsiveness; vROs express claudin-5 and show inner blood-retinal barrier like features, practical and controllable fusion workflow.	Underlying molecular mechanisms remain unclear; true perfusion is not achieved; cost and complexity.
Inagaki et al. [60]	hiPSC	Vascular endothelial cells and pericytes were obtained from mature VOs and mixed with hiPSCs at a ratio of 1:4 at the very start of differentiation.	Enhanced expression of the markers of maturity; enhanced vascularization promotes growth and size; superior model of disease with vascular involvement, eg. diabetic retinopathy.	With a stringent success criterion of >7% of total area as CD31-positive (vascular) area, success rate for obtaining vROs is variable; complex, technically demanding and labor-intensive protocol.
Drabbe et al. [61]	iPSC	At day 30, ROs transferred to an open-well, PDMS-free microfluidic chip that could hold 55 individual ROs for long-term culture, imaging, and retrieval; culture maintained with continuous microfluidic perfusion and vertical oxygen gradient across ROs after day 30.	Better RGC preservation; physiological oxygen microenvironment; extended culture possible beyond 150 days; method allows individual tracking, live imaging, and retrieval for further processing; lesser media use and higher throughput.	Uncertainty about the orientation of ROs during quantification; environment does not fully replicate the in vivo conditions; complex hardware dependence.

EB: Embryoid body; hESC: Human embryonic stem cells; hiPSC: Human induced pluripotent stem cells; mESCs: mouse embryonic stem cells; mPSCs: mouse pluripotent stem cells; SFEBq: Serum-free floating culture of EB-like aggregates; VEGF: Vascular endothelial growth factor

A comparison of ROs shows that optic cups developing from hESC are much larger and neural retina is thicker than those from mouse ESC (mESC). Neural retina in optic cups derived from hESC curves spontaneously in an apically convex manner similar to that in vertebrate retina during development of eye, however, this is not the case with mESC. Furthermore, multilayered neural retina derived from hESC contains both rods and cones, whereas in mESC culture, cone differentiation is poor. These observations may further reflect species differences, given that the mouse retina is rod-dominated, whereas the human retina has a higher proportion of cones [63, 64]. Additionally, mESC-derived ROs reach maturity much faster than those derived from hESC. In one of the studies, development to optic cup formation was shown to take ∼24 days with hESC compared to ∼9 days with mESC, which reflects differences in gestation periods between humans and mice [9]. It has also been observed that genes related to fatty acid metabolism and mitochondrial functions are significantly upregulated and those related to glycogen catabolism and activin receptors are downregulated over time in hPSC-derived ROs. These trends, however, are opposite in mPSC-derived ROs. This may be related to the higher cone density and metabolic adaptations in human-derived ROs, as cones contain significantly more mitochondria than rod photoreceptors [65].

## Structural and functional peculiarities of retinal organoids – current status

An ideal RO should demonstrate developmental fidelity recapitulating human retinogenesis trajectories. It should represent key retinal features such as structural organization with accurate lamination and polarity, diverse cellular composition with all major cell types and synaptic and photoreceptor activity showing functional maturity. It should be reproducible, scalable, and stable ensuring experimental robustness. For assessment of the quality of ROs, evaluation of structure and functionality specific benchmarks have been widely employed (Table 2).

**Table 2:** Benchmarks for assessing retinal organoid quality

Quality parameter	Benchmark	Representative Assays	References
Developmental fidelity	Time-coordinated activation of specific transcription factors corresponding to patterning of the retinal structure.	Expression of LHX2 (earliest to show eye field formation); OTX2 (corresponds to early neural induction); RX (definitive eye field marker essential for OV formation); PAX6 (a master control gene both in NR & RPE for tissue patterning); VSX2 (specific for NR, corresponds to committed RPC and mature bipolar cells).	Kuwahara et al. [25]; Sridhar et al. [27]; Zhong et al. [21]
Polarity & morphology	Apico–basal polarity; presence of outer limiting membrane	Immunostaining (IHC) for apical (ZO-1) and basal (laminin) markers; transmission electron microscopy (TEM)	Gonzalez-Cordero et al. [32]; Capowski et al. [10]
Structural organization in layers	ONL-, INL-, and GCL-like layers	Histological staining such as hematoxylin & eosin; optical coherence tomography, IHC for layer specific markers	Nakano et al. [9]; Kuwahara et al. [25]; Browne et al. [66]
Cellular composition	Presence of seven major retinal cell types (Rods, cones, RGCs, amacrine, Müller glia, bipolar, Horizontal cells).	IHC, single-cell RNA sequencing, flow cytometry	Zhong et al. [21]; Cowan et al. [26]
Photoreceptor maturation	Rod/cone subtype specification; formation of inner segments, outer segments and calyceal processes.	IHC for rhodopsin, S/M/L-opsins for cone specificity, rod outer segment membrane protein 1 (ROM1)	Gonzalez-Cordero et al. [32]; Matsushita et al. [55]
RGC survival	Sustained RGC presence over time	IHC for Brn3A/Brn3B expression over the duration of culture	Wahlin et al. [34] Capowski et al. [10]
Synaptic connections	Functional ribbon synapse formation in the plexiform layers	IHC for markers of ribbon synapses (synaptophysin, C-terminal binding protein 2 (CtBP2)/RIBEYE); TEM for structure of ribbon synapses	Deng et al. [67]; Wahlin et al. [34]
Functional maturity	Light sensitivity and hyperpolarization responses	Calcium imaging, multi-electrode array (MEA); electroretinography	Zhong et al. [21]; Hallam et al. [37]; Cowan et al. [26]
Metabolic support	Metabolic shift to oxidative phosphorylation, reduced necrosis, long-term viability	Metabolic profiling, mitochondrial imaging, hypoxia markers, assays to detect apoptosis	DiStefano et al. [31]; Kim et al. [44]
Reproducibility	Low batch-to-batch variability	Embryoid body size control and use of optimized staging methods	Capowski et al. [10]; Cowan et al. [26]
Scalability	Suitability for screening or modeling	Compatibility for culture in multi-well setting, automation readiness	Mellough et al. [24]; Cowan et al. [26]

Although, no single protocol has been shown to meet all benchmarks simultaneously, currently used protocols generate ROs that possess most of the retinal cell types including RPE cells, photoreceptors (both rods and cones), RGCs, horizontal, bipolar, amacrine and Müller cells in a multilamellar organization after a prolonged culture using various protocols [68]. Identification of interneurons including bipolar, horizontal and amacrine cells is difficult due to their low numbers, however, Müller glial cells, survive well with typical morphology spanning the entire neuroretina in hPSC-derived ROs [10].

In one of the studies, a diverse cohort of 16 hPSC lines were cultured for at least 175 days to understand morphological development of ROs. It was observed that development of ROs can be differentiated into three stages. In the first stage ROs have a developing outer neuroblastic layer containing numerous RPCs, and inner retinal layer containing RGCs with very few amacrine cells. The inner plexiform-like layer is discontinuous and rudimentary. In stage two, RPCs differentiate into photoreceptors, horizontal and amacrine cells while RGC layer gradually deteriorates. In stage 3, photoreceptors mature and develop outer segments. Development of Müller glia and formation of external limiting membrane is also observed at this stage. Notably, while the outer retinal organization becomes advanced, there is continued loss and disorganization of inner retinal cells and layers with prolonged culture. By day 150 of culture, presence of two layers of rudimentary but functional synapses could be observed; these include i) outer plexiform connections where photoreceptors form ribbon synapses contacting bipolar and horizontal cells, and ii) inner plexiform connections consisting of bipolar cells synapsing with RGCs [10]. In the hiPSC-derived ROs, light responses could also be evoked in 16.7% of outer nuclear cells and 12.0% of inner nuclear and ganglion cells. Further, it was noted that photoreceptors in these ROs hyperpolarize in response to light stimuli, suggesting that the inner organoid cells, which respond downstream, may predominantly exhibit characteristics of OFF neuronal circuitires [26]. In contrast, another study demonstrated ‘ON’-type responses in RGCs within hPSC-derived ROs, reporting a 25% increase in spiking activity following exposure to white light pulses, indicating functional diversity and partial recapitulation of retinal circuitry [42].

A comparison of human organoids with fetal retina has shown remarkable similarity in cellular composition, though some differences are obvious. Organoids on day 60 closely resembled fetal retina at equivalent developmental stage (day 59). Nonetheless, although both contained similar cell types, the proportion of cells differed with greater number of RPC and lesser number of RGCs compared to fetal retina. Organoids also lack non-neuronal cells such as endothelial cells, astrocytes and microglia that have important roles in cell proliferation, survival, and other developmental processes including synapse formation [27]. Importantly, ROs lack structural and functional RPE-photoreceptor relationship and interphotoreceptor matrix that is crucial for development and survival of neural retina. In ROs, RPE is present at one pole of the organoids away from neural retina. Liu et al. have described a protocol to obtain mature RPE cell enriched spheroids that could serve as a source of expandable RPE cells along with neural retina in hiPSC-derived ROs [69]. This study also could not observe the desired RPE-photoreceptor relationship. Besides the absence of retinal vasculature and choriocapillaris, the lack of key RPE functions such as phagocytosis, regeneration of photoreceptor outer segments, recycling of phototransduction pigments, and nutrient exchange also limits organoid development. Additionally, the loss of neurotrophic and vasculotropic factors, which normally support growth and survival, combined with the absence of an appropriate extracellular matrix, creates an unfavorable microenvironment. Together, these factors hinder the full maturation, structural organization, and functional development of retinal components compared to in vivo conditions [70].

Single-cell RNA sequencing (scRNA-seq) analyses comparing mouse native retinae and iPSC-derived ROs have shown that, while ROs broadly mimic native developmental trajectories, notable differences remain. For instance, photoreceptor precursor cells tend to emerge prematurely and dominate the cellular composition of ROs [71]. Despite such differences, several retinopathy-associated genes exhibit conserved expression profiles between human retinae and murine ROs, suggesting partial molecular fidelity. To benchmark the developmental accuracy of human ROs, Kim et al. compared RO-derived transcriptomes with fetal and adult human retinal atlases [72]. By identifying key markers of retinal cell types and developmental stages, they assessed multiple differentiation protocols and highlighted those that yielded the highest resemblance to native human retinae. More recently, an integrative approach combining scRNA-seq and spatial transcriptomics revealed that the spatial and temporal dynamics of cell type specification in ROs closely mirror human retinogenesis, underscoring the potential of optimized organoid systems for modeling human retinal development and disease [73]. Hence, ROs provide much advanced experimental setting compared to animal models and 2-D cell culture systems to study retinal diseases and therapeutics. Among various cell types, photoreceptors and RGCs are the most well characterized in ROs.

### Photoreceptors

In ROs, compared to other retinal cell types, photoreceptors retain significant cellular architecture. In humans, retinogenesis begins in central retina and then maturation progresses towards peripheral retina [74]. This central to peripheral maturation supports the formation of cone-rich central macula and a perifoveal region with higher rod density. A central pit in the macula, the fovea centralis, is responsible for high visual acuity due to its dense L/M cone population. The photoreceptor population in para- and perifoveal regions is a mixed one, containing both cones and rods. Noticeably, photoreceptors constitute nearly 80% of retinal cells in mice and humans. Rods outnumber cones by 35:1 in mice while rod to cone ratio in humans is 18–20:1 [63, 75]. In ROs, the photoreceptor composition of neuroretina is perifoveal-like with a higher number of rods than cones. The late stage (>30 weeks) transcriptomes from organoids strongly correlate with adult human peripheral retina [10, 26, 32]. ROs consistently lack the anatomical features of the macula, including the foveal region where L/M cones are normally concentrated in the human retina. Instead, rod and cone subtypes are more uniformly distributed throughout the outer nuclear layer, indicating a lack of regional specialization and fovea-like organization of human retina.

Several studies have shown that early progenitors of photoreceptors appear around day 80–120 of culture. Subsequently, from day 120–180, brush-border-like structures corresponding to the photoreceptor inner and outer segments develop [10]. Kaya et al. have described a protocol that utilizes 9-cis instead all-trans retinoic acid for accelerated differentiation of rods in hPSC-derived RO [43]. A higher rhodopsin expression and mature mitochondrial morphology was evident by day 120 of culture. The development of photoreceptors can be tracked using biomarkers specific to each maturation stage. For example, in early differentiation stage, RPC factor, VSX2, and field transcription factor, PAX6, are expressed. Subsequently, CRX, a cone photoreceptor precursor-specific transcription factor, and NRL, an early rod-specific marker, are upregulated. Recoverin and L/M/S-opsins and rhodopsin then appear as markers of the mature cones and rods, respectively. PCARE protein staining from day 120–180 marks the presence of photoreceptor cilium and outer segment formation [76]. The presence of disks in photoreceptor outer segments attached to mitochondria-rich inner segments via connecting cilia has been reported at ultrastructural level in ROs. Moreover, presence of ribbon synapses between photoreceptor axons and second-order neuron was also observed [22, 26, 34, 44, 77].

In order to investigate pathologies and potential therapeutics primarily involving photoreceptors, photoreceptor-enriched organoids have been generated through modification of standard protocols. A 2-D/3-D protocol, in which retinoic acid concentration was reduced between day 50–70, promoted differentiation of iPSCs to mature photoreceptors with rudimentary outer segments that were light-responsive [21]. Development of photoreceptors with rudimentary outer segments could also be facilitated by adding differentiating retinal factors such as serum, retinoic acid, taurine, and the supplements N2 and B2732 [26]. Another protocol developed by Lowe et al. cultured clumps of Matrigel adherent hESC to obtain single lumen epithelialized cysts. Following the formation of epithelialized cysts, adherent culture promoted the development of retinal monolayers, which, when transferred to a floating culture, gave rise to ROs. After long-term culture, these ROs formed stratified retinal tissue containing abundant photoreceptors that exhibited outer segment-like ultrastructures [29]. The 3-D ROs developed from confluent cells avoiding EB formation were also shown to have rich population of mature rods and cones that could be cryopreserved while retaining their phenotypic characteristics for further use. [33, 35, 36] West et al. have also described a protocol where antioxidants and fatty acids supplementation facilitates formation of photoreceptor outer segments with organized stacked discs in both rods and cones in mature hPSC-derived ROs. [53] In a combined 2-D/3-D differentiation system, hPSC-derived ROs were shown to consist of photoreceptors with structural and molecular characteristics suitable for transplantation [32, 78].

Notably, a desirable proportion of cones and rods may be achieved by protocol adaptations that modulate specific signaling pathways. For example, addition of docosahexaenoic acid (DHA) was shown to promote higher expression of rhodopsin in rods, while FGF1 supported biogenesis and maintenance of cones in mouse ROs [40]. In PSC-derived ROs, generation of rods and S-cones was enhanced by addition of retinoic acid and triiodothyronine (T3) during days 90 – 120 of differentiation; L/M-cones generation was enhanced at the expense of rods by combined addition of retinoic acid from days 30 – 120 and a γ-secretase inhibitor ((2S)-N-[(3,5-Difluorophenyl)acetyl]-l-alanyl-2-phenyl]glycine1,1-dimethylethyl ester2L) from days 28 – 42 of differentiation; generation of S-cones was enhanced at the expense of rods by addition of levodopa and retinoic acid from days 90 – 120 of differentiation [48]. Addition of retinoic acid at later stages of differentiation was shown to favour the generation of mature ROs from hiPSCs with a predominant rod population [52]. In another study by Li et al., ROs derived from hiPSC obtained from urine were cultured without addition of retinoid acid at any stage of differentiation. Under these culture conditions, up to 50% of the ROs showed a predominant rod photoreceptor population while 43% were L/M cone rich. However, the population of S-cones was sparse [39].

Investigations focusing on central blindness resulting from foveal cone death such as retinal dystrophies, including macular degenerative diseases require cone-rich experimental setting. For this reason, protocols for the generation of cone-rich organoids have been attempted [79, 80]. Generation of cone-enriched ROs requires specific protocol modifications to direct subtype-specific cone differentiation. One key factor is thyroid hormone (TH) signaling, which plays a crucial role in regulating cone subtype fate through the TH receptor beta (THRβ). Low TH signaling during early development favors S-cone fate, while high TH signaling at later stages promotes L/M-cone specification. Continuous exposure of ROs to active T3 throughout the photoreceptor differentiation period has been shown to significantly shift cone identity from S- to M-subtypes [35]. Using such refined protocols, cone-enriched ROs have been successfully generated and characterized. In one study, the enrichment of cones after 8 months of culture was confirmed through single-cell RNA sequencing [44]. Notably, in another study, a substantial fraction of cones across multiple organoids derived from different hPSC lines displayed robust light-evoked electrical responses, with membrane properties comparable to those of cones in ex vivo primate fovea [81].

### Retinal ganglion cells

In vivo as well as in ROs, RGCs are the first cells to develop. In vivo, a graded expression of various chemoattractant and/or chemorepellent molecules and transcription factors derived from RPE and other sources along with intracellular signaling guides their axonal projections into the optic nerve. However, it is noticeable that in ROs, RPE is not juxtaposed to the outer nuclear layer of photoreceptors and develops in the form of adjacent clumps. As a result, there is a lack of diffusible factors from RPE that direct RGCs differentiation in ROs, adding to the lack of neurotrophic factors originating from visual cortex. Therefore, a progressive and stochastic loss of RGCs on long-term culture was a key limitation in early ROs studies. Additionally, lack of trophic support due to loss of RGCs also causes loss of interneurons followed by remodeling [26, 27]. However, to advance research focused on RGCs and their synaptic interactions with interneurons, it is critical to develop ROs capable of sustaining robust RGC survival over extended culture periods to serve as a renewable source of RGCs for cell replacement therapies and disease modeling. One hallmark feature of mature RGCs is the extension of long neurites, which in vivo correspond to axons projecting out to reach precise post-synaptic targets within the brain’s visual pathways. This axonal projection capacity reflects the intrinsic biophysical properties and synaptic integration capabilities of RGCs highlighted in physiological studies, emphasizing the importance of replicating these features in vitro to closely model visual circuitry [82, 83]. Current studies have now optimized the in vitro conditions to prolong RGC survival. A study by Fligor et al., showed that laminin, a key component of the ECM in the nerve fiber layer significantly enhances RGC neurite outgrowth in ROs. The addition of Netrin-1 further enhanced the number and average length of neurites [36]. Among the 11 different types of laminins mainly laminin-332 and laminin γ3 expression have been observed in the neural retina during retinogenesis and these expressions are conserved in ROs derived from PSCs. Laminin γ3 in particular was recognized to be important for laminar organization or human retina, organisation of photoreceptors, RGC differentiation and synapse formation [84]. Netrin-1 is expressed in optic nerve head astrocytes and guides the growth of RGC axons out into the optic nerve [85]. Hence, the use of laminin and Netrin-1 allows RO RGCs to closely represent in vivo retinogenesis and early developmental stage of RGC axonal outgrowth. Fligor et al. identified 5 groups of RGCs in ROs each expressing a unique axon guidance receptor gene [36]. It was proposed that this heterogeneity of RGC population may be due to cells undergoing different stages of development or may signify differences in postsynaptic targets. Another study described a substantial number of RGCs bearing two surface markers CD184 and CD171 in the basal layer of hiPSC-derived ROs during 40–60 days of culture. These RGCs survived and integrated in mice with normal and optic nerve crush [86]. In most of these protocols, about 40–50 days are needed for generating RGCs. Wagstaff et al. have described a method that combines free-floating culture protocol with the 3-D Matrigel culture to drastically reduce the culture time to generate ROs [50]. With this protocol, a significant number of neuronal cells staining positively for RGC specific markers could be generated in 28 days and outgrowth of RGCs was sustained till 90 days of culture [50].

## Retinal organoids – current applications

ROs provide a valuable system for studying human retinal diseases, which range from inherited conditions like RP to complex diseases such as AMD. These diseases result in vision loss due to the dysfunction or death of RPE and retinal neurons. While therapies such as anti-angiogenic agents for wet AMD [87] and gene therapy for Leber congenital amaurosis (LCA) [88] have shown promise, their benefits are limited, and they are far from being curative or universally effective.

Use of ROs has particularly been useful in understanding the developmental biology of the retina and the pathogenesis of retinal diseases. ROs allow researchers to study cellular interactions and the effects of genetic mutations in a controlled environment [89, 90]. For instance, organoids derived from patients with specific genetic mutations have been used to investigate early developmental abnormalities, providing insights into the mechanisms underlying diseases like RP and Usher syndrome [91, 92]. Modeling diseases such as glaucoma and AMD is difficult due to extremely complex aetiologies and significant involvement of factors such as aging. VanderWall et al. have described a glaucoma model for which the OPTN(E50K) mutation, a leading cause of inherited glaucoma, was introduced into existing lines of hPSCs using CRISPR/Cas9 gene editing and isogenic controls were generated from patient-derived lines [93]. The data suggested that the pathogenesis of inherited glaucoma could be recapitulated in RGCs in ROs. This research is vital for identifying potential therapeutic targets and understanding how genetic variations affect retinal development and function. Moreover, advancements in bioengineering techniques are enhancing the potential of ROs for therapeutic applications. The development of tissue-engineered scaffolds and the integration of ROs with other cellular systems are being explored to improve cell survival and integration post-transplantation [94, 95]. These innovations aim to develop safer and more effective treatments for retinal degeneration by addressing key challenges including transplant rejection and tumorigenicity that may be associated with hiPSC-derived therapies [96, 97].

### Modeling mendelian forms of retinal degeneration

Over 300 genes and genetic loci have been linked to human retinal diseases, ranging from inherited orphan diseases to complex conditions involving multiple genes. Many of these genes are crucial for retinal development, and loss of their function leads to early-onset symptoms in childhood or even at birth [98, 99]. Common retinal diseases include LCA, RP, and cone-rod dystrophy (CRD), all of which are characterized by the abnormal development or function of photoreceptors. LCA is a leading cause of childhood blindness, with 14 known genes implicated, while RP and CRD have also been linked to numerous genetic mutations, affecting millions worldwide.

hPSC-derived ROs offer a powerful model for studying these heritable retinal diseases. With advanced genetic editing technologies like CRISPR/Cas9, researchers can introduce or correct mutations in stem cell lines to study their effects on retinal cells [100–102]. By using such approaches to introduce mutations into well-characterized stem cell lines, using patient-derived iPSCs, or repairing mutations in these cells, the disease-specific and patient-specific effects could be determined for personalized treatment approaches. In fact, early studies have shown that patient-derived organoids reveal critical defects in photoreceptors such as in RP and LCA, aiding the understanding of genotype-phenotype relationships and the development of targeted therapies [103]. In a notable study by Tucker, iPSCs derived from the patient’s keratinocytes were differentiated into eyecup-like structures that resembled human RPCs [103]. These structures exhibited photoreceptor markers and features such as axonemes and basal bodies characteristic of outer segments. Analysis revealed that one mutation caused a frameshift and premature stop codon, while the Arg4192His mutation led to protein misfolding and endoplasmic reticulum stress, as shown by increased GRP78 and GRP94 levels [103]. When these cells were transplanted into mice, they formed recognizable photoreceptors, indicating that the mutations contribute to post-developmental photoreceptor degeneration [103].

Using iPSCs from patients to create ROs has proven valuable for studying inherited retinal ciliopathies. The primary cilia of photoreceptors, crucial for phototransduction, can be affected by genetic defects in cilia-associated proteins. These defects are linked to several hereditary retinal diseases, including LCA, RP, Bardet-Biedl syndrome, and Senior-Loken syndrome. Modeling these ciliopathies in mice has been challenging due to differences in affected cell types and retinal degeneration rates compared to humans. Parfitt et al. were among the first to use ROs derived from iPSCs of an LCA patient’s fibroblasts to study photoreceptor cilia [77]. In this study, the researchers used iPSCs with a common *CEP290* mutation to create differentiated photoreceptors and RPE in 3-D optic cups. The iPSCs differentiated into RPE and optic cups as expected, despite issues with *CEP290* splicing and cilia defects [77]. The most severe splicing abnormalities and cilia defects were found in optic cups, which explains the retina-specific effects of the mutation. Treatment with an antisense morpholino corrected the splicing issue, restored full-length *CEP290* expression, and normalized cilia function [77]. This pioneering study has demonstrated the potential of hPSC-derived ROs for tracking disease progression and evaluating treatments for genetically-based retinal degeneration.

Deng et al. [67] investigated the impact of a specific mutations using ROs. They obtained iPSCs from three RP patients with different frameshift mutations in the *RPGR* gene, which were then differentiated into ROs with RPE cells possessing electrophysiological properties. Significant defects were observed in photoreceptor morphology, localization, transcriptional profiles, and electrophysiological activity, along with shortened cilia in both the patient iPSCs and organoids [67]. Correcting the *RPGR* mutation using CRISPR-Cas9 restored photoreceptor structure, improved electrophysiological function, reversed ciliopathy, and normalized gene expression. In fact, in this study [67] Deng and co-authors demonstrated the use of patient-specific organoids to model *RPGR*-related pathogenesis and provided proof-of-concept for targeted gene therapy. Similarly, disrupted alternative splicing in specific splicing programs, with mis-splicing of genes encoding pre-mRNA splicing proteins was detected only in patient-specific retinal cells and *Prpf31*± mouse retinas and RPE [104]. Mis-splicing in genes related to ciliogenesis and cellular adhesion led to severe RPE defects, including disrupted apical-basal polarity, reduced trans-epithelial resistance and phagocytic capacity, and decreased cilia length and occurrence. Disrupted cilia morphology was also observed in patient-derived photoreceptors, leading to progressive degeneration and cellular stress. In situ gene editing of the pathogenic mutation restored protein expression and corrected key cellular phenotypes in RPE and photoreceptors [104]. It has also been observed that reduced PRPF31 levels correlate with disorganized RPE cells and impaired phagocytosis. Unlike transgenic mouse models, these organoids accurately replicated the human phenotype, showcasing rod photoreceptor degeneration followed by cone cell death. Correcting the pathogenic mutation via CRISPR/Cas9 restored normal morphology, suggesting haploinsufficiency as a potential cause of retinal disease [105].

Studying late-onset retinal degeneration (L-ORD) like AMD and glaucoma using in vitro systems is challenging due to a complex interplay of genetic and environmental factors. These diseases often involve unknown genetic mutations that contribute to patient susceptibility, making it difficult to model individual risk accurately. One approach is to use patient-derived organoids with high-risk alleles and exposing them to disease-related stresses for identification of specific cell types prone to damage or death. This could help generate a profile of genetic and environmental risk factors, aiding disease modeling and the development of preventive strategies.

Although modeling these diseases is difficult, ROs still hold promise for predicting patient risk, studying disease progression, and testing therapies. For example, research has shown that patient-derived iPSCs with specific risk alleles may produce retinal cells that are less mature or more prone to degeneration, offering insights into potential biomarkers for early disease detection. Example of such approach is the study of Teotia et al. [106] which utilized iPSCs derived from glaucoma patients carrying the *SIX6* risk allele to generate functional RGCs. Findings revealed that iPSCs with the *SIX6* risk allele produced RGCs less efficiently than those from healthy controls, showing developmental abnormalities such as shorter, simpler neurites and an immature electrophysiological profile. Additionally, these RGCs exhibited increased expression of glaucoma-related genes, indicating an early onset of disease characteristics [89].

Another study by Gao at al. developed a RO model from patient-derived iPSCs with a *PDE6B* mutation, associated with RP. In fact, this research presents the first consistent in vitro model of late-onset RP and provides new insights into the role of *PDE6B* in the disease. Initially, the patient-derived ROs showed normal retinal development up to day 180, but by day 230, abnormal localization of rods was observed. The authors concluded that this may be related to cGMP accumulation, which was detected from day 193 and peaked at day 230, with higher cGMP levels in ROs compared to controls. This accumulation could potentially disrupt synaptic connections and the connecting cilium in photoreceptors [62].

A major challenge in modeling L-ORD is that age is a significant risk factor, but organoids do not naturally replicate the aging process within a typical laboratory timeframe. To address this, researchers are exploring methods to accelerate aging in vitro. To improve the relevance of ROs for modeling age-related retinal diseases, Sridhar et al. reviewed innovative approaches that simulate aging within these in vitro systems [27]. One of these involved the ectopic expression of progerin, a mutant form of lamin A that accumulates in Hutchinson-Gilford progeria syndrome, leading to premature cellular aging. When introduced into ROs, progerin induces molecular and structural features consistent with aged retinal cells. Another strategy entailed direct conversion of somatic cells from elderly donors into induced retinal cells without passing through a pluripotent state. This method preserved age-associated epigenetic and transcriptional signatures that are typically erased during iPSC reprogramming. These approaches enhance the capacity of ROs to recapitulate aging phenotypes, making them more suitable for studying the mechanisms of age-related retinal degeneration and for testing potential interventions [89].

### Pharmacological evaluation and toxicological drug screening

ROs, derived from hPSCs, have emerged as a transformative tool in pharmacological evaluation of drugs for retinal diseases. The ability to generate ROs from patient-specific iPSCs enables researchers to study disease mechanisms and evaluate potential treatments tailored to individual genetic backgrounds [107]. For instance, studies have shown that organoids derived from patients with RP exhibit disease-specific features, making them ideal for testing targeted therapies [62]. This personalized approach not only enhances the relevance of drug testing but also aligns with the growing emphasis on precision medicine in ophthalmology [89].

A significant advancement using ROs is demonstrated in a study by Saengwimol et al., which tested the effects of chemotherapeutics on retinoblastoma models [108]. The researchers developed 3-D organoids from chemotherapy-naïve tumors and assessed their drug responses, comparing them to those observed in advanced, recurrent retinoblastoma. The organoids exhibited key histological features and genetic profiles of retinal tumors, including cone signal circuitry and a glial tumor microenvironment. Topotecan, alone or in combination with melphalan, effectively targeted proliferative tumor cells, while methotrexate was less effective. Notably, drug responses in these organoids mirrored clinical outcomes, demonstrating the reliability of patient-derived organoids for testing new retinoblastoma therapies. Another study by Srimongkol et al. evaluated the antineoplastic agent sunitinib in patient-derived retinoblastoma organoids [109]. The results showed that sunitinib effectively reduced cell viability in a dose-dependent manner with minimal toxicity, highlighting its potential as a therapeutic option. A range of assays was used to assess cell viability, proliferation, and apoptosis, confirming the utility of organoids in modeling tumor responses to chemotherapy [109].

In addition to cancer studies, ROs have been used to explore the effects of developmental modulators. For example, retinoic acid was shown to delay initial photoreceptor differentiation, resulting in more structured and mature organoids [52]. In another experimental study, blocking CXCR4 led to cellular mislocalization in the organoids, indicating the compound’s influence on cell migration and tissue polarity during retinal development [110]. Use of ROs has also been evaluated for toxicological screening. In one of the studies, hPSC-derived ROs when exposed to six known retinotoxic compounds (ketorolac, digoxin, thioridazine, sildenafil, ethanol, methanol) showed photoreceptor cell death, Müller glia activation, and reduced RGC responsiveness to light. These effects closely correlated with in vivo toxicity profiles for corresponding drugs and hence validated the use of ROs as a drug screening platform [111]. Although these preliminary studies have shown promise, the successful use of organoids in drug discovery remains challenging, especially in achieving consistent, large-scale results.

### Gene therapy

Gene therapy using ROs derived from hiPSCs represents a rapidly advancing field with significant implications for treating retinal diseases. The main research directions in this area focus on improving the efficacy of gene therapy, understanding retinal disease mechanisms, and developing innovative therapeutic strategies. By using ROs, researchers can replicate the effects of genetic mutations on photoreceptors, providing a human-platform to evaluate the potential of gene therapies before moving to clinical trials. For instance, organoids allow for the precise delivery of gene therapy vectors, such as adeno-associated viruses (AAVs), and enable detailed assessments of how these treatments restore gene function, protein expression, and cellular structure. Additionally, ROs have demonstrated utility for evaluating the long-term effects and potential side effects of gene therapies, particularly AAV-based strategies. ROs can serve as a powerful platform for optimizing photoreceptor transduction efficiency through capsid engineering and promoter modifications, enabling the fine-tuning of AAV vectors to target specific retinal cell types effectively [112–114]. However, a significant limitation of current organoid models is the absence of an immune system, which means they cannot replicate innate or adaptive immune responses that often accompany intraocular delivery of viral vectors such as inflammatory reactions, neutralizing antibody production, or cytotoxic T cell activation. Consequently, while ROs are excellent for assessing cellular tropism and promoter strength, they cannot predict immunogenicity or inflammation risks, which still must be evaluated in vivo using animal models [115]. Pioneering studies have explored various aspects of gene therapy and optogenetic technologies in ROs. For example, Garita-Hernandez et al. investigated the use of optogenetic technologies in ROs derived from hiPSCs as a model to evaluate the membrane trafficking of microbial opsins for potential therapeutic applications in retinal diseases [116]. They tested both depolarizing and hyperpolarizing opsins (CatCh, ChrimsonR, ReaChR, eNpHR 3.0, and Jaws) to determine their efficiency in membrane localization. Their findings indicated that eNpHR 3.0, ReaChR, and Jaws exhibit the highest membrane localization due to enhanced trafficking signals, while CatCh and ChrimsonR showed less efficient membrane targeting, leading to protein accumulation in the endoplasmic reticulum and Golgi, resulting in endoplasmic reticulum stress and an unfolded protein response. This study highlights that hiPSC-derived ROs are effective screening tools for predicting the subcellular behavior of optogenetic proteins in human retinal cells, supporting their use in validating gene therapy products and drug molecules [116].

Quinn et al. further elucidated the role of ROs in gene therapy by demonstrating their utility in modeling RP. Their study focused on the CRB1 gene [113]. The authors showed that hiPSC-derived ROs can effectively recapitulate the disease phenotype, allowing for the assessment of AAV-mediated gene therapy targeting both photoreceptors and Müller glia [113]. This dual targeting is essential for achieving therapeutic efficacy, as it addresses the complex cellular environment of the retina. The findings also highlighted the importance of selecting optimal AAV serotypes and promoters to maximize transgene expression in the relevant cell types, thereby enhancing the potential for successful gene therapy outcomes [113]. McClements et al. contributed to this body of work by investigating the tropism of AAV vectors in photoreceptor-like cells within ROs [114]. Their research indicates that different AAV serotypes exhibit varying degrees of efficiency in transducing these cells, which is crucial for the development of effective gene therapies. By systematically evaluating the transduction capabilities of various AAV vectors, the study provides valuable insights into the design of gene therapy protocols that can be tailored to specific retinal cell types. The studies conducted by Guo et al. [91] and Kruczek et al. [117] significantly advance the understanding of the application ROs in gene therapy for retinal diseases, particularly focusing on the modeling of RP and LCA. Guo et al. explored the potential of hiPSC-derived ROs to model RP, specifically investigating the impact of the *USH2A* mutation [91]. *USH2A* mutations are commonly associated with Usher syndrome type II, a disorder characterized by moderate-to-severe congenital hearing loss and progressive vision loss due to RP. This study demonstrates that ROs generated from patients with this mutation exhibit early developmental abnormalities, which are crucial for understanding the disease pathogenesis. The study provides insights into the cellular and molecular mechanisms underlying the disease, thereby identifying several key therapeutic targets such as cilium-associated genes (*CFAP43* and *PIFO*) central to photoreceptor maintenance and implicated in Usher syndrome, synaptic genes (*DLGAP1* and *GRIK1*) involved in retinal connectivity and signal transmission, and genes related to apoptosis and stress-response pathways (*CASP3* and *HIF1A*). Kruczek et al. focused on the application of gene therapy in ROs derived from patients with dominant CRX-associated LCA [117]. They demonstrate the feasibility of using AAV-mediated gene therapy to rescue photoreceptor function in these organoids. The study highlights the importance of understanding the specific genetic mutations associated with LCA, as different mutations can lead to varying degrees of retinal degeneration.

To optimize dosage and cell specificity for gene replacement therapies, Na et al. [112] conducted both qualitative and quantitative analyses of transgene expression in ROs using these AAV2 and AAV8 serotypes. While no neurotoxicity was observed, AAV2 showed higher transduction efficiency in Müller glial cells compared to AAV8 and both were similarly effective in rods and cones. This suggested that hPSC-derived ROs could be an effective platform for testing gene therapy efficacy, cell type specificity, and toxicity. Additional studies have focused on enhancing gene therapy efficacy through AAV capsid, transgene promoter, and inverted terminal repeats modifications, with ROs serving as an initial screening platform that complements animal models by bridging basic research and translational medicine.

Another recent example is the study by Sladen et al. [118], where researchers generated isogenic RP GTPase regulator (*RPGR*) knockout (KO) iPSCs via CRISPR/Cas9 gene editing and differentiated these *RPGR*-KO and wild-type iPSCs into ROs to test AAV-*RPGR* clinical vector construct [118]. Transduction of *RPGR*-KO ROs with AAV-*RPGR* successfully restored *RPGR* mRNA and protein expression, localizing it to the photoreceptor connecting cilium in both rod and cones [118]. The vector-derived *RPGR* exhibited glutamylation levels equivalent to those in wild-type ROs. Additionally, AAV-*RPGR* treatment corrected rhodopsin mislocalization in *RPGR*-KO ROs, reducing its presence in the photoreceptors. A key limitation of the study is the absence of the RPE in the organoids, which affects the analysis of AAV tropism. To address this, more complex models have been developed, such as RO-brain assembloids [119] and retina-on-a-chip [120–122]. However, significant challenges remain, especially regarding tropism and the interaction between the RPE and the neural retina.

### Cell therapy

For cell therapy, ROs have emerged as a promising source for retinal transplant, particularly in retinal degeneration. In fact, the field of retinal transplantation has evolved from simple injections of cultured cells [123] to complex tissue engineering, due to notable shifts in methodologies. The current landscape for cell therapy in retinal degenerative conditions, like RP, is defined by two primary transplantation formats, cell suspensions and retinal sheets.

Cell suspensions involve the delivery of dissociated, single cells or small aggregates, typically via standard subretinal injections and their use is described in various studies [80, 124–127]. In contrast, use of intact retinal sheets consisting of 3-D neuroepithelial grafts that preserve the native laminar architecture of the ROs has also been utilized in several studies [128–133] (Table 3). The use of cell suspension has been shown to provide the functional recovery mainly due to material transfer (cytoplasmic exchange) rather than replacement of host cells by donor cells [124]. In contrast, use of 3-D retinal sheets derived from ROs could structurally and functionally integrate into end-stage degenerate retinas and restore light-responsive behavior by forming new synaptic circuits. In fact, the transition from simple survival to the optimization of synaptic connectivity is a central theme in recent literature, which emphasizes that high-acuity vision restoration depends on the precision of surgical delivery and the specific maturation stage of donor cells [137].

**Table 3:** Comparative overview of the transplantation outcomes using 3-D retinal sheets vs. dissociated cell suspensions in retinal degeneration

Transplanta-tion Format	Recipient/Model	Key Outcomes (Integration & Survival)	Functional Mechanism & Trade-offs	Representative Studies
Cell suspension (Dissociated/single cells)	*rd1* and *rho-/-* mice; RCS rats; 13-lined ground squirrel; Mice with NMDA-induced retinal injury.	Faster delivery; shorter-term survival (3–4 weeks to 3 months). High incidence of material transfer (cytoplasmic exchange) noted by Pearson et al.	**Mechanism:** Primarily paracrine support or material transfer in retinal degeneration models; true integration is limited. **Trade-off:** Simplified delivery but lower structural fidelity.	Yu et al. [80] Tucker et al. [127] Pearson et al. [124] Lei et al. [125] Lin et al. [126] Ripolles-Garcia et al. [134] Zhao et al. [135]
Retinal sheets (Intact 3-D grafts)	*rd1* and *rho-/-* mice; *Cynomolgus* monkeys; Rat models; Human clinical trial (retinitis pigmentosa).	Robust survival (up to 2 years in humans). Formation of host-graft synapses and organized outer nuclear layers. Risks of “rosettes” formation if not properly placed.	**Mechanism:** True cell replacement and structural reconstruction of the retinal architecture. **Trade-off:** High functional potential for end-stage disease but requires complex surgical intervention.	Assawachananont et al. [131] Mandai et al. [132] McLelland et al. [133] Hirami et al. [130] Iwama et al. [129] Watanabe et al. [128] Shirai et al. [136]

Studies included in this table highlight a strategic divergence in the field. Early work and recent RGC research favor cell suspensions for their ease of delivery. However, studies across multiple species, from rodents to primates and finally humans consistently demonstrate that the sheet format is superior for reconstructing the laminar architecture required for high-acuity vision. The future of these formats lies in the precision of ‘incorporating these 3-D structures into the host circuitry via genome editing and advanced surgical maneuvers.

A direct comparison of these approaches reveals distinct trade-offs. Cell suspensions offer a simplified delivery approach with lower surgical trauma but often result in a disorganized integration profile and limited survival due to the lack of structural support. Retinal sheets, while requiring highly specialized surgical tools and more invasive procedures, provide a superior structural scaffold that promotes true cell replacement and the formation of host-graft synaptic circuits. While suspensions provide paracrine-mediated rescue in early disease, retinal sheets are increasingly viewed as capable of restoring function in end-stage degeneration where the host architecture is lost.

Recent technical advances have refined the outcomes by using genome-edited ROs. To improve the host-graft connectivity, these ROs can be optimized by removing graft bipolar cells, ensuring that signals are processed directly through the host’s innate visual pathways [128]. While the majority of retinal transplantation research focuses on degenerative photoreceptor loss, some studies have expanded the scope to include retinal structural defects and acute injuries affecting other cellular components. One of the examples is the work by Iwama et al. [129], which investigated the use of retinal sheets harvested from hPSC-derived ROs to treat refractory macular holes, representing a physical break in the foveal tissue unlike degenerative diseases that have cell loss but intact retinal architecture. A successful mechanical closure of the macular hole with improved fixation and light sensitivity was observed in this study after transplanting retinal sheets directly into the foveal center in a primate model. Another significant diversion from standard photoreceptor replacement is the targeting replacement of lost RGCs, such as in glaucomatous optic neuropathy. Lei et al. [125] utilized dissociated RGC precursors enriched from human iPSC-derived ROs and transplanted them into mice with NMDA-induced acute retinal injury. These transplanted precursors developed into mature RGCs, integrated into the GCL and extended neurites within three weeks. Similarly, researchers have explored models of RPE dysfunction, such as the Royal College of Surgeons (RCS) rat used by Lin et al. [126], to determine if RO-derived sheets can provide functional rescue when the underlying metabolic support layer of the retina is the primary site of pathology.

The landmark clinical study by Hirami et al. [130] represents the first successful long-term application of these technologies in humans. As a representative of the retinal sheet transplantation format, this study transplanted clinical-grade allogeneic iPSC-derived retinal sheets into the macular or perimacular area of two patients with advanced RP. Key findings included a two-year confirmed survival and stable integration without any signs of immune rejection, teratoma formation, or serious adverse events. The results also showed localized preservation of retinal structure. Achievements such as stabilized fixation points near the graft area suggested a localized improvement in cone function. Despite these structural successes, significant challenges remain, including a lack of direct evidence for synapse formation in the host and the absence of detectable electrophysiological responses in clinical tests. Although, this study seems to bridge the gap between pre-clinical animal models and human regenerative medicine; its limitations such an open label study design, involvement of only 2 patients with no control groups and limited evaluations require further investigations. A co-transplantation of RPE with ROs to support the metabolic retinoid cycle and the survival of photoreceptor outer segments is an important future strategy to address the major limitation of severe lack of RPE in advanced RP.

Overall, a comparative methodological evaluation across studies highlights distinct trade-offs between biological simplicity and functional efficacy. Dissociated cell suspensions offer the benefit of easier surgical delivery through standard needles but may have challenges in terms of low integration efficiency. Furthermore, the extent and effectiveness of “material transfer” remains uncertain. Conversely, 3-D retinal sheets provide superior structural scaffold and a higher potential for true cell replacement, but they require highly specialized surgical tools and more invasive procedures to reach the subretinal space. Furthermore, autologous iPSCs (patient-specific) eliminate immune rejection risks and the allogeneic “off-the-shelf” approach used by Hirami et al. [130] is significantly more practical for large-scale clinical application, provided that immune stability can be maintained. The emerging use of genome-edited and scaffold-supported grafts aims to overcome these barriers by specifically engineering the tissue to “plug” into host circuitry more efficiently. Ultimately, as highlighted by Donato et al. [137], translating these sophisticated animal successes into human clinical results requires a multi-disciplinary approach that tailors the surgical format to the specific pathological environment of the degenerate host retina.

## Retinal organoids: challenges in mimicking the native retina

Although ROs derived from PSCs can recapitulate many features of human retinal development, they fall short of fully mimicking the complex structure and functions of the native retina.

### Structural limitations

Key architectural limitations include the lack of a layered, laminated structure, incomplete synaptic connectivity, and improper juxtaposition between photoreceptors and the RPE. In ROs, the RPE often forms clumps instead of a monolayer adjacent to photoreceptors, critical for photoreceptor maintenance and visual cycle function. Furthermore, vascular and immune components, including microglia, are absent, limiting physiological relevance and leading to diffusion-restricted necrosis of inner organoid layers in long-term culture. The spherical morphology of ROs exacerbates this issue, reducing nutrient and oxygen penetration.

### Functional and synaptic limitations

ROs exhibit partial light responsiveness due to the presence of some functional ON and OFF pathways and rudimentary ribbon synapses between photoreceptors and bipolar/horizontal cells. However, essential synaptic specializations such as ribbon synapses at bipolar cells in the inner plexiform layer and synapses specific to rod or cone pathways are either absent or underdeveloped. As a result, ROs cannot fully replicate signal processing functions related to contrast, colour, motion, and depth perception. This limited synaptic integration significantly constrains their use for advanced functional studies or drug screening aimed at neural circuit restoration.

### Variability and technical barriers

Heterogeneity among ROs, often resulting from differences in cell lines, differentiation protocols, and culture conditions is also a significant challenge. ROs at varying developmental stages can coexist in the same culture, complicating data interpretation and limiting reproducibility. Additionally, prolonged culture time and low yield hinder scalability for high-throughput applications. Nonetheless, recent improvements such as the use of microwell arrays for uniform EB formation and checkerboard scraping techniques have significantly increased the homogeneity and yield of ROs, with some protocols producing thousands of ROs per well [26, 56].

## Emerging strategies and technological innovations

To overcome the structural and functional limitations of conventional RO models, researchers are developing advanced bioengineering strategies that enhance nutrient delivery, physiological maturation, and recapitulation of in vivo-like retinal microenvironments [39]. These innovations include bioreactors [138, 139] and oxygen-permeable materials to improve nutrient and gas exchange, co-culture systems with mesodermal and vascular progenitors [140], and scaffold engineering using biomimetic extracellular matrices [141] to support more physiological tissue architecture. Significant advances have emerged from retina-on-a-chip platforms and 3-D bioprinting approaches, which integrate microfluidic control, vascularization, and multi-cellular complexity. Retina-on-chip systems combine hiPSC-derived retinal cells or organoids with microfluidic channels to create perfusable microenvironments that mimic retinal physiology, capturing multi-cellular interactions and tissue-level functions to improve disease modeling and therapeutic testing [142]. These engineered chips can impose physiologically relevant oxygen gradients, supporting inner and outer retinal layer maturation. For example, Drabbe et al. [61] designed a RO chip that maintains defined oxygen gradients, enhancing RGC viability and addressing limitations of static cultures. Microfluidic perfusion further improves nutrient delivery, waste removal, and organoid longevity.

3-D bioprinting complements these approaches by enabling the precise construction of retinal tissue architectures. Using tailored bioinks, layered fabrication, and scaffold design, researchers can assemble retinal cells alongside supporting matrices and vascular components in defined patterns, improving nutrient diffusion, cell–cell interactions, and long-term tissue survival [143]. Advanced applications include 3-D printed microfluidic devices for RO-endothelial co-culture, which facilitate the formation of perfusable choroid-like vascular networks integrating retinal structures. These models replicate vascular features relevant to diseases such as wet age-related macular degeneration, providing platforms for drug testing, gene therapy evaluation, and disease modeling.

The integration of retina-on-chip [39], 3-D bioprinting [144], and co-culture strategies further enhances vascularization and tissue maturation. Bioprinting allows precise placement of retinal cells, including photoreceptors, Müller glia, and ganglion cells, alongside vascular cells such as endothelial cells and pericytes within a supportive extracellular matrix. Co-culture introduces additional cell types, including mesodermal progenitors and RPE, promoting cell–cell signaling, tissue polarization, and functional maturation. Together, these strategies enhance synaptic connectivity, photoreceptor outer segment formation, and electrophysiological activity, producing organoids that more faithfully replicate in vivo retinal structure and function [120–122]. These integrated platforms are particularly valuable for personalized gene therapy development. Patient-derived hiPSCs can generate disease-specific organoids in which AAV serotypes, promoters, and vector constructs are systematically evaluated. Combining gene therapy with advanced organoid, retina-on-chip, and vascularized bioprinted platforms overcomes structural and functional limitations of conventional organoids, bridging the gap between in vitro studies and clinical translation. Integration with CRISPR/Cas9-based gene editing further enables precise modeling of inherited retinal diseases and assessment of therapeutic strategies in a patient-specific context.

Although retina-on-chip, 3-D bioprinting, and co-culture approaches improve nutrient delivery, vascularization, and layer organization, ROs remain relatively immature compared to the adult human retina. To bridge this gap, long-term maturation strategies, incorporating extended culture periods, oxygen optimization, and supportive microenvironments, are critical to promote photoreceptor outer segment formation, synaptic connectivity, and electrophysiological activity. Extending culture duration beyond early differentiation stages (e.g., >130–200 days) has been shown to promote more complete laminar organization, elongated photoreceptor outer segments, and enhanced visual pigment accumulation, resulting in more physiologically mature ROs with improved electrophysiological properties compared with shorterterm cultures [145]. Another example, novel 3-D printed stirred bioreactors maintain physiological oxygen levels and significantly improve RO yield, viability, and reproducibility by preventing hypoxiainduced necrosis in deeper organoid layers, thereby enabling extended maturation [146]. Similarly, microfluidic RO chips that maintain stable oxygen gradients support inner and outer retinal cell survival during extended culture (>150 days), facilitating the maturation of RGCs and photoreceptor phenotypes [61]. Additional strategies such as modifying the extracellular environment, like adding hyaluronan, a component of the interphotoreceptor matrix, can accelerate photoreceptor commitment and promote outer segment development, further enhancing long-term maturation [57].

Beyond microenvironmental optimisation, re-establishing physiologically relevant intercellular interactions is a critical next step toward achieving mature and functional human retinal models. Retina–RPE co-culture systems, often referred to as retina–RPE assembloids, represent an important advancement in promoting long-term maturation and functional fidelity of ROs. By physically and functionally integrating hiPSC-derived ROs with RPE monolayers or organoids, these systems restore essential epithelial–neural interactions that are lacking in isolated ROs. Such interactions support photoreceptor outer segment elongation, retinoid cycling, metabolic exchange, and outer segment phagocytosis, leading to more adult-like photoreceptor phenotypes and improved electrophysiological responses during extended culture periods (>150–200 days). Recent studies demonstrate that retina–RPE assembloids enhance photoreceptor survival, promote visual pigment expression, and improve synaptic organization, thereby better recapitulating in vivo retinal structure and function [26, 39, 105]. These platforms are increasingly integrated with microfluidic and oxygen-controlled systems to further support long-term maturation and enable more predictive disease modeling and therapeutic testing.

## Translational feasibility and regulatory considerations

Despite their promise for disease modelling and therapy development, translating RO–derived products into experimental and clinical practice remains challenging due to manufacturing, reproducibility, safety, scalability, and regulatory constraints. First, GMP-grade (or cGMP-aligned) production remains a bottleneck. Many RO protocols depend on animal-derived matrices (e.g., Matrigel), undefined supplements, and manual selection steps, all of which complicate process validation, lot-to-lot consistency, and traceability. Current translational strategies emphasize replacing xenogeneic components with xeno-free, chemically defined media and matrices, closed or semi-closed workflows, and in-process quality control metrics that can be standardized across sites. Reviews specifically discussing translation of RO production toward GMP standards highlight the need for qualified raw materials, defined release criteria, and process controls suitable for regulated manufacturing (e.g., identity, purity, potency, sterility, and genomic stability assays) [147].

Second, variability across iPSC lines (donor genetic background, epigenetic memory, reprogramming method, passage number) can lead to meaningful differences in RO cell composition, maturation, and phenotype, directly impacting clinical reproducibility and product comparability. This is increasingly recognized as a core translational risk: even when morphology appears similar, transcriptomic and functional endpoints may diverge, necessitating multi-layered quality control (e.g., scRNA-seq benchmarking, functional electrophysiology where relevant, and standardized marker panels) and robust comparability plans for manufacturing changes [148].

Third, immunological and safety considerations are central for any RO-derived cell or tissue product. Although the eye is relatively immune-privileged, immune activation can occur particularly with allogeneic products, vector delivery, or surgical trauma. Safety programs typically require demonstrating: (i) absence of undifferentiated pluripotent cells (tumorigenicity risk), (ii) genetic stability (karyotype monitoring, sequencing-based checks), (iii) lack of adventitious agents, and (iv) acceptable biodistribution/ectopic tissue risk in vivo. For photoreceptor replacement specifically, literature emphasizes that outcomes depend strongly on donor cell maturation state, cell preparation format (sheet vs suspension), surgical delivery variables, and evidence of synaptic integration, all of which must be controlled and documented for clinical translation [149].

Fourth, translation requires scalability: therapeutic manufacturing demands consistent output at clinically relevant doses and cost. Scale-up approaches (automation, bioreactors, controlled aggregate size, closed culture) can improve throughput but introduce new process-related risks (shear stress, diffusion gradients, altered differentiation trajectories), reinforcing the need for potency-linked release assays and stability programs. Experience from GMP-oriented organoid manufacturing in other tissues supports feasibility but also illustrates the extensive process development needed to convert research workflows into validated production pipelines [150].

Finally, regulatory classification and approval pathways constitute a complex and jurisdiction-dependent challenge. Across regulatory systems, RO-derived therapies are generally regarded as high-risk advanced products, reflecting their degree of manipulation, biological complexity, and intended therapeutic use. In the United States, most RO-derived products are unlikely to qualify for regulatory exemptions applicable to “361 human cells, tissues, and cellular and tissue-based products (HCT/Ps),” as they typically exceed minimal manipulation and do not meet homologous-use criteria under 21 CFR Part 1271. Consequently, RO-derived therapies are expected to be regulated as biological products, requiring full preclinical development, investigational new drug submission, and phased clinical trials, making FDA guidance on biologics classification directly relevant [151].

In Europe, RO-derived therapies would almost universally fall under the Advanced Therapy Medicinal Products (ATMP) framework, necessitating GMP-compliant manufacturing, extensive quality and comparability documentation, validated potency and safety assays, and robust clinical evidence aligned with European Medicines Agency expectations [152]. Following Brexit, the United Kingdom has retained a largely parallel ATMP framework, with independent oversight by the Medicines and Healthcare products Regulatory Agency and comparable regulatory requirements [153]. In Russia, stem cell and organoid-derived therapies are regulated as biomedical cell products under Federal Law No. 180-FZ, mandating state registration, GMP manufacture, preclinical testing, and formal clinical trials prior to clinical use [154].

Across Asia, regulatory approaches remain heterogeneous but are converging toward international standards. In China, stem cell- and cell-based therapies are governed by a dual regulatory system, whereby investigator-initiated clinical research operates under national stem cell clinical research measures with ethical approval and record filing, while commercial development is regulated as biological drugs under the Drug Administration Law and overseen by the National Medical Products Administration [155, 156]. In India, RO-derived therapies would be classified as new drugs under the New Drugs and Clinical Trials Rules, with oversight by the Central Drugs Standard Control Organization and compliance with national stem cell research guidelines issued by the Indian Council of Medical Research, including requirements for ethical approval, GMP-aligned manufacturing, and structured clinical trial progression [157]. In Southeast Asia, regulatory pathways remain less harmonised but increasingly align with FDA and EMA principles, with organoid-derived products generally treated as high-risk cell-based therapies requiring early regulatory engagement and case-by-case assessment.

## Conclusions and future perspectives

ROs have established themselves as a powerful 3-D in vitro system that recapitulates key aspects of human retinogenesis, including the sequential emergence of major retinal cell types, layered organization, and partial functional maturation. By bridging the gap between traditional 2-D cultures and animal models, ROs provide a uniquely human-relevant platform for studying retinal development, elucidating disease mechanisms, and interrogating genotype–phenotype relationships in inherited and complex retinal disorders. Their ability to be derived from patient-specific pluripotent stem cells further enhances their value for personalized disease modeling and therapeutic evaluation.

Despite these advances, current RO systems remain constrained by several limitations that are consistently highlighted across studies, including incomplete laminar organization, progressive loss of inner retinal neurons, limited long-term functional maturation, lack of vascular and immune components, and substantial inter- and intra-line variability. These challenges restrict the direct translation of ROs into fully predictive models of adult retinal physiology and underscore the need for continued methodological refinement. Importantly, no single differentiation protocol currently satisfies all structural, functional, and scalability benchmarks simultaneously, reinforcing the necessity of protocol selection tailored to specific experimental objectives.

Encouragingly, recent innovations integrating bioengineering approaches, such as microfluidic perfusion, vascularized organoid fusion, controlled oxygen and metabolic environments, and co-culture with non-neuronal cell types, are progressively improving tissue viability and physiological relevance. In parallel, advances in genome editing, single-cell and spatial transcriptomics, and standardized benchmarking frameworks are enhancing the reproducibility, interpretability, and translational relevance of RO-based studies. Together, these developments are positioning ROs as increasingly robust platforms for drug discovery, toxicological screening, and evaluation of gene- and cell-based therapeutic strategies.

Looking ahead, ROs are likely to serve an expanding role as complementary experimental systems within the retinal research and therapeutic development pipeline. While direct clinical application of intact organoids remains at an early stage, RO-derived cell populations are already informing translational strategies, and further optimization may enable broader regenerative applications. Continued integration of biological insight, engineering innovation, and regulatory awareness will be essential to fully realize the potential of ROs as tools for advancing retinal biology and developing more effective therapies for blinding diseases.

## Data Availability

No datasets were generated or analysed during the current study.
